# Glutamine and Its Precursors Supplementation Improve Growth Performance and Immunity and Regulate Gastrointestinal Microbiota of Suckling Lambs

**DOI:** 10.3390/life16061012

**Published:** 2026-06-16

**Authors:** Wenjie Zhang, Feier Ren, Zhonghao Wang, Weibing Zhang, Kai Feng, Yulong Zhao, Hailiang Wang, Hongyan Hou, Shiyin Wang, Wei Zhang

**Affiliations:** Key Laboratory of Livestock and Poultry Healthy Breeding Technology in Northwest China, College of Animal Science and Technology, Xinjiang Agricultural Vocational and Technical University, Changji 831100, China; xjauzwj@126.com (W.Z.); xjnzdrfe@163.com (F.R.); wzhh1402@163.com (Z.W.); zhangweibingvip@163.com (W.Z.); 15292414396@163.com (K.F.); xjnydx_zyl@163.com (Y.Z.); hlwangup@163.com (H.W.); hhy1402@163.com (H.H.)

**Keywords:** α-ketoglutarate, antioxidant capacity, glutamic acid, glutamine, growth performance, gut microbiota, immune function, suckling lamb

## Abstract

This study systematically compared the effects of dietary supplementation with glutamine (Gln) and its precursors, including glutamic acid (GA) and α-ketoglutarate (AKG), on growth performance, serum antioxidant and immune parameters, and multi-region gastrointestinal microbiota in suckling lambs. Forty healthy suckling Hu lambs with similar body weight (7.37 ± 1.18 kg) and age (7 ± 0.8 d) were selected and randomly allocated into four groups (*n* = 10 per group): a control group (CON, without additive), and three treatment groups (GA, AKG, and Gln), each receiving 2 g per animal per day of the corresponding additive. The experimental period lasted for 42 d. All three additives showed a tendency to increase the final body weight (*p* = 0.056) and significantly increased the average daily gain (ADG) of lambs (*p* < 0.05). GA supplementation increased the dry matter intake throughout the entire trial (*p* < 0.05), whereas the addition of AKG and Gln increased the dry matter intake only during the later period (d 21–42) (*p* < 0.05). The feed-to-gain ratios did not differ among all groups (*p* > 0.05). Compared with the CON group, all three treatment groups showed elevated serum activities of catalase, glutathione peroxidase, and total antioxidant capacity, as well as increased IgA and IgG contents (*p* < 0.05). In addition, malondialdehyde concentration was decreased in all three treatment groups (*p* < 0.05). Moreover, GA supplementation reduced the ruminal alpha diversity while increasing the abundance of butyrate-producing bacteria (*Ruminococcaceae UCG-014*) (*p* < 0.05). All three interventions consistently decreased the abundance of the intestinal pathogen *Escherichia-Shigella* in the ileum (*p* < 0.05). Correlation analyses showed that ruminal *Treponema 2* abundance was negatively correlated with ADG, whereas jejunal *Methylobacterium* and ileal *[Eubacterium] coprostanoligenes group* were positively correlated with final body weight or ADG. In conclusion, glutamine and its precursors play an important role in modulating gastrointestinal bacterial diversity and composition, enhancing antioxidant and immune functions, and improving the growth performance of suckling lambs.

## 1. Introduction

As an important source of high-quality animal protein, mutton occupies a significant position in global animal husbandry. According to statistics from the Food and Agriculture Organization of the United Nations (FAO), global lamb meat production and consumption have continued to rise over the past two decades, particularly in Asia, Africa, and the Middle East, where the sheep meat sector has become an important pillar for food security and farmers’ income generation [1,2]. However, compared with industries such as dairy cattle and swine, meat sheep production generally exhibits lower efficiency, with one core bottleneck being the high preweaning mortality and slow early growth of lambs. It is estimated that preweaning lamb mortality ranges from 10% to 30% globally and is even higher in some extensively managed regions [3,4,5]. These early losses not only represent substantial economic waste but also severely constrain the sustainable development of the meat sheep industry [6,7]. The causes of lamb mortality and growth retardation are complex and multifactorial, including maternal malnutrition, inadequate management, environmental stressors, and infectious disease; however, the fundamental physiological factor is the immature development of the neonatal gastrointestinal tract [8]. Newborn lambs have weak intestinal barrier function, insufficient digestive enzyme secretion, and unstable microbial colonization, rendering them highly susceptible to invasion by pathogens such as *Escherichia coli* and *Salmonella*, which can lead to diarrhea, metabolic disorders, impaired immunity, and consequently growth arrest or death [9]. Therefore, improving lamb survival rates and promoting early growth performance have become urgent scientific problems and practical challenges for the international sheep industry.

Unlike adult ruminants, the abomasum of neonatal lambs is the largest of the four stomach compartments and serves as the primary organ for nutrient digestion and absorption. After suckling, esophageal groove closure directs milk straight into the abomasum, where milk forms curds under the action of abomasal enzymes and is subsequently digested and absorbed slowly in the small intestine [8,10]. With increasing age and the transition from milk dependence to solid feed, the rumen gradually develops, microbial colonization commences, and volatile fatty acids promote maturation of the ruminal epithelium; concurrently, the small intestine, as the key absorptive site, has its absorptive efficiency directly determined by mucosal villus height, crypt depth, and the integrity of epithelial tight junctions [11]. Moreover, the gut represents the largest immune organ of the body, with approximately 70% of immune cells residing in gut-associated lymphoid tissues. Therefore, promoting intestinal development, optimizing gut microecology, and enhancing immune function in young ruminants are critical strategies for improving early health and growth performance in lambs.

Glutamine (Gln) is the most abundant conditionally essential amino acid in the body and serves as a primary energy substrate for intestinal epithelial cells, lymphocytes, and macrophages. Extensive research indicates that Gln plays a central role in maintaining intestinal mucosal integrity, promoting epithelial proliferation and repair, and enhancing intestinal barrier function [12]. In studies of young animals, Gln has been shown to reduce the incidence of post-weaning diarrhea in piglets and to promote villus development [13]. In growth-retarded yaks, Gln has likewise demonstrated effects in promoting small intestinal development [14]. However, poor stability and low solubility of Gln can lead to cyclization in aqueous solutions or during feed processing and storage, producing compounds such as pyroglutamate and ammonia [15], which may have potential adverse effects and thus limit its practical application.

Gln can be deaminated to generate glutamic acid (GA), which is further converted to α-ketoglutaric acid (AKG); both AKG and GA can serve as precursors that combine with ammonium to resynthesize Gln [12]. Therefore, supplementation with Gln precursors (GA and AKG) may confer physiological functions similar to or even superior to direct Gln supplementation, while offering improved stability and safety. Recent studies indicate that glutamine and its precursors extend beyond mere energy provision, encompassing modulation of the gut microbiota, antioxidant effects, and immune regulation. Regarding the gut microbiota, Gln can increase the abundance of beneficial bacteria (e.g., *Lactobacillus* and *Bifidobacterium*) and inhibit colonization by pathogens (e.g., *Escherichia coli*); the mechanisms may involve regulation of intestinal pH, alteration of mucin secretion, and modification of bacterial adhesion sites [16,17]. In terms of antioxidant activity, Gln is a precursor for glutathione (GSH) synthesis and, via activation of the Nrf2 signaling pathway, enhances activities of antioxidant enzymes such as SOD and GSH-Px, reduces MDA levels, and mitigates oxidative stress-induced damage [18,19,20]. For immune modulation, Gln provides energy for lymphocyte proliferation and intestinal sIgA secretion, regulates the balance between proinflammatory cytokines (TNF-α and IL-6) and anti-inflammatory cytokines (IL-10), and enhances host immune defenses [21,22,23]. Although glutamine and its precursors have been investigated relatively systematically in monogastric animals (suckling pigs, broiler chickens), studies in young ruminants, particularly suckling lambs, remain very limited. Existing reports have largely focused on Gln itself, lacking direct comparisons with precursors such as GA and AKG; moreover, no systematic evaluations have been reported that concurrently assess the effects of these three compounds on lamb gut microbiota composition, antioxidant capacity, and immune function. Given the pronounced differences between ruminants and monogastrics in digestive physiology, intestinal developmental trajectories, and microbial colonization patterns, directly extrapolating findings from monogastric species may entail risks. Therefore, a systematic evaluation of the effects of Gln and its precursors on growth performance, gut microbiota, antioxidant status, and immune capacity in suckling lambs holds significant theoretical value and practical importance for production guidance.

## 2. Materials and Methods

### 2.1. Animal Ethics

All experimental animal care and handling procedures in this study were conducted in accordance with the Guidelines for the Care and Use of Laboratory Animals in China and were approved by the Animal Care Committee of Shihezi University of China (protocol permit number: A2025-887).

### 2.2. Experimental Design

The Hu sheep is a renowned local breed in China, well known for its high fecundity, early sexual maturity, strong adaptability, and fine meat quality. Hu ram lambs grow relatively fast and are suitable for meat production systems. This breed was chosen because of its economic importance in intensive lamb production and its representative value for studying early-life nutritional interventions. The experiment was conducted from July 2025 to September 2025 at Huikang Animal Husbandry Farm, Changji, Xinjiang, China. Forty healthy, suckling Hu ram lambs with an initial body weight of 7.37 ± 1.18 kg and age of 7 ± 0.8 days were selected and randomly assigned to four treatment groups (*n* = 10 per group) using a random number generator (http://www.r-project.org/, accessed on 1 July 2025). One group served as the control (CON) and received only milk replacer (procured from Beijing Precision Animal Nutrition Research Center, China). The remaining three groups were designated Gln, GA, and AKG; in addition to the same milk replacer, each lamb in these groups received a daily supplement of 2 g of glutamine (Gln), glutamic acid (GA), or alpha-ketoglutarate (AKG), respectively (all additives purchased from Chengdu Baishixing Technology & Industry Co., Ltd., Chengdu, China). The Gln dosage (2 g/d) was determined based on preliminary experiments from our laboratory and the study by Sciascia et al. [24] in suckling piglets; for comparability, the GA and AKG dosages matched that of Gln. Milk replacer was mixed with water at a 1:7 ratio. Feeding volumes increased with age as follows: phase 1 (days 1–10), 300 mL/day/lamb; phase 2 (days 11–20), 450 mL/day/lamb; phase 3 (days 21–30), 600 mL/day/lamb; phase 4 (days 31–42), 400 mL/day/lamb. Milk replacer was offered three times daily at 09:00, 14:00, and 19:00, with the additive incorporated into the morning (09:00) feeding as a single dose. All experimental lambs had ad libitum access to starter feed (procured from Xinjiang Taikun Feed Co., Ltd., Changji, China) from 7 days of age; the composition and nutrient levels of the starter are presented in Table 1. The experimental period lasted 42 days, including a 2-day adaptation period followed by a 40-day formal trial; weaning was carried out for all lambs at the end of the formal trial.

### 2.3. Feeding and Management of Experimental Lambs

All experimental lambs were ear-tagged and housed individually in single pens (1.5 m × 1.5 m) within the same barn. The barn featured raised beds, and each pen was equipped with a feeding trough and a drinker to ensure uniform housing conditions for all lambs. During the trial, lambs had ad libitum access to clean drinking water; the temperatures of both the milk replacer and the drinking water were maintained between 32 and 36 °C to ensure palatability and minimize stress. Vaccination schedules and pen disinfection procedures were implemented in strict accordance with the farm’s routine management protocols.

### 2.4. Determination and Analysis of Experimental Lamb Parameters

#### 2.4.1. Growth Performance

Daily solid feed intake was recorded. Weigh all lambs before the morning feeding on day 0 (pre-trial) and on days 10, 20, 30, and 42, and calculate average daily gain (ADG) from initial and final body weights. Feed intake was determined as the difference between offered and refused feed and converted to dry matter intake (DMI). Feed conversion ratio (F:G) was calculated as actual DMI divided by ADG.

#### 2.4.2. Blood

On the morning of day 40 of the trial, prior to feeding, eight lambs were randomly selected from each treatment group for blood sampling. Using vacuum blood-collection tubes without anticoagulant, 5 mL of blood was drawn from the jugular vein of each lamb. The samples were then centrifuged at 3500× *g* for 15 min at 4 °C to obtain serum, which was aliquoted into 1.5 mL Eppendorf tubes, labeled and stored at −20 °C for subsequent assays of blood parameters.

Serum biochemical parameters, including total protein (TP), albumin (ALB), total cholesterol (TC), triglycerides (TG), blood urea nitrogen (BUN), alkaline phosphatase (ALP), aspartate aminotransferase (AST), alanine aminotransferase (ALT), and glucose (GLU), were measured using a fully automated biochemical analyzer (ZYKHB-1280; Huaren Biotechnology Co., Ltd., Nanjing, China).

Serum antioxidant parameters, including activities of catalase (CAT), superoxide dismutase (SOD), and glutathione peroxidase (GSH-Px), total antioxidant capacity (T-AOC), and malondialdehyde (MDA) were measured using assay kits (Nanjing Jiancheng Bioengineering Institute, China; catalog numbers: A007-1-1, A001-3-2, A005-1-2, A015-2-1, A003-1-2) in the laboratory of Xinjiang Agricultural Vocational and Technical University.

Serum immune parameters, including immunoglobulin G (IgG), immunoglobulin A (IgA), immunoglobulin M (IgM), interleukin-6 (IL-6), interleukin-1β (IL-1β), interleukin-10 (IL-10), and tumor necrosis factor-α (TNF-α), were measured using kits (Shanghai Enzyme-linked Biotechnology Co., Ltd., Shanghai, China; catalog numbers: ml109391, ml025601, ml025607, ml025540, ml058059, ml824689, ml025652) in the laboratory of Xinjiang Agricultural Vocational and Technical University.

#### 2.4.3. Ruminal, Jejunal, and Ileal Digesta

On experimental day 42, prior to morning feeding, six suckling lambs were randomly selected from each group and euthanized by intravenous injection of sodium pentobarbital (50 mg/kg body weight). The rumen, jejunum, and ileum were rapidly excised using cotton twine and scissors; digesta from each of these compartments were homogenized, aliquoted into 10 mL RNase-free cryovials, and immediately snap-frozen in liquid nitrogen. Samples were reserved for determination of bacterial composition and diversity in the rumen, jejunum, and ileum.

#### 2.4.4. 16S rRNA Sequencing and Bioinformatics Analysis of Bacterial Communities in the Rumen, Jejunum, and Ileum

Total genomic DNA from ruminal, jejunal, and ileal digesta samples was extracted using the cetyltrimethylammonium bromide (CTAB) method [25]. Bacterial DNA concentration and purity were assessed by 1% agarose gel electrophoresis and spectrophotometry. Primers 338F (5′-ACTCCTACGGGAGGCAGCA-3′) and 806R (5′-GACTACHVGGGTWTCTAAT-3′) were designed to amplify the V3–V4 variable region of the bacterial 16S rRNA gene. PCR amplification conditions were as follows: initial denaturation at 98 °C for 1 min; denaturation at 98 °C for 10 s; annealing at 54 °C for 30 s; extension at 75 °C for 30 s, for 30 cycles in total; and a final extension at 72 °C for 5 min. Paired-end libraries were constructed using the TruSeq DNA PCR-Free Sample Preparation Kit, and library quality was evaluated with a Qubit@2.0 fluorometer (Thermo Fisher Scientifi, Shanghai, China). After passing quality control, libraries were sequenced on an Illumina HiSeq 2500 platform (Illumina, San Diego, CA, USA). Raw 16S rRNA gene sequencing data were filtered using FLASH (v1.20) and QIIME (v1.9.1) to obtain high-quality effective sequences [26]. Uparse (v7.0.1001) was employed to cluster OTUs and to select the most frequent sequence within each OTU as the representative sequence for downstream annotation [27]. Taxonomic annotation was performed using the Mothur method against the SILVA SSU rRNA database with default confidence values (0.8–1) [28], and microbial community composition was analyzed at the phylum, family, and genus levels for each sample. Alpha and beta diversity indices were calculated using QIIME (v1.9.1). Differentially abundant taxa among intestinal digesta sites were identified using LEfSe with a default LDA score threshold of 4.

### 2.5. Data Analysis

Preliminary analyses of the experimental data were conducted using Excel 2010. Before performing one-way ANOVA, the assumptions of normality of residuals and homogeneity of variances were checked. Specifically, residuals from the ANOVA model were tested for normality using the Shapiro–Wilk test, and homogeneity of variances was assessed using Levene’s test. Both assumptions were met for all data. One-way ANOVA was then performed using SPSS 22.0 (SPSS Statistics 22, IBM Japan, Ltd., Tokyo, Japan). Data are presented as mean ± standard deviation; when significant differences were detected, multiple comparisons were performed using Duncan’s test. A threshold of *p* < 0.05 was used to indicate statistical significance, and 0.05 < *p* < 0.10 was considered indicative of a trend. Pearson correlation analysis was used to evaluate associations between production performance and differential genera in the rumen, jejunum, and ileum, and figures were generated with Origin 8.0 (OriginLab Co., Northampton, MA, USA).

## 3. Results

### 3.1. Effects of Glutamine and Its Precursors on the Growth Performance of Suckling Lambs

As shown in Table 2, compared with the CON group, the GA, AKG, and Gln groups exhibited higher final body weight (*p* = 0.056, indicating a trend) and significantly higher average daily gain in lambs (*p* < 0.05). Over the entire experimental period, DMI in the GA group was significantly increased relative to CON (*p* < 0.05); DMI in the AKG and Gln groups showed a significant increase compared with CON during days 21–42 (*p* < 0.05). No significant differences in feed conversion ratio (F:G) were observed among the treatment groups (*p* > 0.05).

### 3.2. Effects of Glutamine and Its Precursors on Serum Immunoglobulin Levels in Suckling Lambs

As shown in Table 3, compared with the CON group, the GA, AKG, and Gln groups exhibited significant increases in IgA and IgG contents (*p* < 0.05). Specifically, for IgA, the GA, AKG, and Gln groups increased by 10.45%, 8.96%, and 8.96%, respectively, relative to the CON group (*p* < 0.05). For IgG, the GA, AKG, and Gln groups increased by 16.35%, 20.20%, and 22.52%, respectively (*p* < 0.05). The IgM content in the GA group was also significantly elevated compared with the CON group (*p* < 0.05), with no significant differences observed between the GA, AKG, and Gln groups (*p* > 0.05).

### 3.3. Effects of Glutamine and Its Precursors on Blood Inflammatory Cytokines in Suckling Lambs

As shown in Table 4, compared with the CON group, IL-1β levels were significantly reduced in the GA and AKG groups (*p* < 0.05); IL-6 levels were significantly reduced in the GA, AKG, and Gln groups relative to the CON group (*p* < 0.05); IL-10 levels in the CON group were significantly higher than those in the AKG group (*p* < 0.05); no significant differences were observed among groups in TNF-α levels (*p* > 0.05).

### 3.4. Effects of Glutamine and Its Precursors on the Antioxidant Capacity of Blood in Suckling Lambs

As shown in Table 5, the SOD activity in the AKG group was significantly higher than that in the CON and Gln groups (*p* < 0.05). Compared with the CON group, the activities of CAT and GSH-Px in the GA, AKG, and Gln groups were significantly increased (*p* < 0.05). The T-AOC in the GA and AKG groups was significantly higher than that in the CON and Gln groups (*p* < 0.05). Compared with the CON group, the MDA content in the GA and AKG groups was significantly decreased (*p* < 0.05), while there was no significant difference compared with the Gln group (*p* > 0.05).

### 3.5. Effects of Glutamine and Its Precursors on Blood Biochemical Parameters in Suckling Lambs

As shown in Table 6, compared with the CON group, the ALB content in the GA, AKG, and Gln groups was significantly increased (*p* < 0.05). There were no significant differences in serum TP, GLB, TC, TG, BUN, ALP, AST, ALT, and GLU contents among the four groups (*p* > 0.05).

### 3.6. Effects of Glutamine and Its Precursors on the Composition and Diversity of Rumen Bacterial Communities in Suckling Lambs

**Figure 1:**
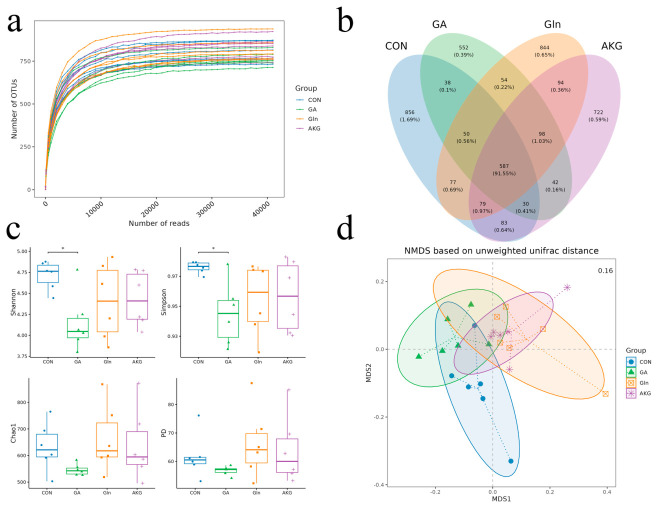
Rarefaction curves (**a**), OTUs (**b**), α-diversity analysis (**c**), and β-diversity analysis (**d**) of ruminal bacteria in suckling lambs from the CON, GA, Gln, and AKG groups. CON: Each lamb was fed only milk replacer daily; GA: Based on the same milk replacer, each lamb received an additional 2 g/day of GA (Chengdu, China); AKG, Based on the same milk replacer, each lamb received an additional 2 g/day of AKG (Chengdu, China); Gln, Based on the same milk replacer, each lamb received an additional 2 g/day of Gln (Chengdu, China). “*” indicates a significant difference between groups where *p* < 0.05.

As shown in Figure 1, with increasing sequencing depth the rarefaction curves for ruminal microbiota in all four groups converge toward a plateau, indicating that most of the microbial diversity was captured. (Figure 1a). As illustrated in Figure 1b, a total of 4206 OTUs were identified across the four groups, of which 587 OTUs were shared, accounting for 13.96% of the total; the numbers of group-specific OTUs were 856 (CON), 552 (GA), 844 (Gln) and 722 (AKG), respectively. Alpha diversity analysis indicated no significant differences in Shannon, Simpson, Chao1, or PD indices between the Gln and AKG groups and the CON group (*p* > 0.05), whereas the GA group exhibited significantly reduced Shannon and Simpson indices relative to CON (*p* < 0.05) (Figure 1c). Beta diversity analysis using PERMANOVA (Bray–Curtis distance, 999 permutations) revealed a significant difference in ruminal microbial community structure among the four groups (R^2^ = 0.12, *p* = 0.028). Pairwise comparisons showed that the GA and AKG groups differed significantly from the CON group (*p* < 0.05), while the Gln group did not (*p* > 0.05) (Figure 1d).

**Figure 2:**
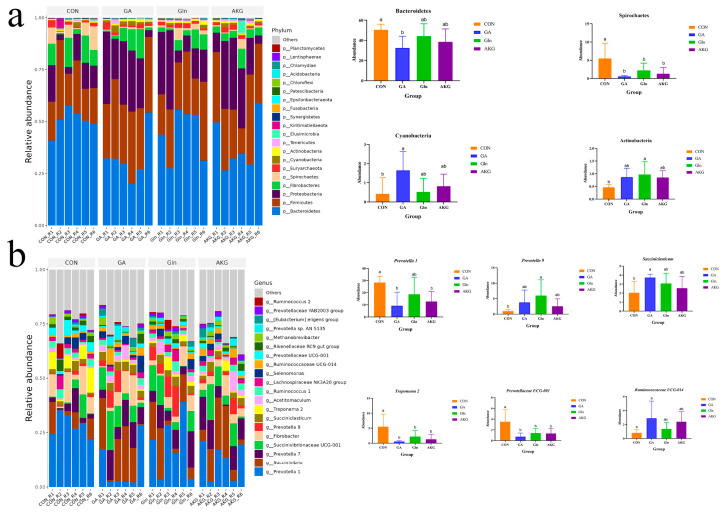
Relative abundance of ruminal bacteria at the phylum level (**a**) and genus level (**b**) in suckling lambs from the CON, GA, Gln, and AKG groups. CON: Each lamb was fed only milk replacer daily; GA: Based on the same milk replacer, each lamb received an additional 2 g/day of GA (Chengdu, China); AKG, Based on the same milk replacer, each lamb received an additional 2 g/day of AKG (Chengdu, China); Gln, Based on the same milk replacer, each lamb received an additional 2 g/day of Gln (Chengdu, China). In the bar charts, values with different letters differ significantly (*p* < 0.05).

At the phylum level, the dominant bacterial communities in the rumen of all groups were Bacteroidetes, Firmicutes, Proteobacteria, and Fibrobacteres, with Bacteroidetes showing the highest relative abundance, followed by Firmicutes. Among these, compared with the CON group, the relative abundance of Bacteroidetes in the GA group was significantly reduced (*p* < 0.05), and the relative abundances of ruminal Spirochaetes in the GA, Gln, and AKG groups were all significantly reduced (*p* < 0.05). The relative abundances of ruminal Cyanobacteria in the GA and AKG groups were significantly increased compared to the CON group (*p* < 0.05). The relative abundances of ruminal Actinobacteria in the GA, Gln, and AKG groups were all increased compared to the CON group, with a significant difference between the Gln group and the CON group (*p* < 0.05) (Figure 2a). At the genus level, the dominant bacterial communities in the rumen of all groups were *Prevotella 1*, *Succinivibrio*, *Prevotella 7*, and *Succinivibrionaceae UCG-001*, with *Prevotella 1* having the highest relative abundance, followed by *Succinivibrio*. Among these, compared with the CON group, the relative abundance of *Prevotella 1* in the GA and AKG groups was significantly reduced (*p* < 0.05), while the relative abundance of *Prevotella 9* in the Gln group was significantly increased compared to the CON group (*p* < 0.05). Compared with the CON group, the relative abundances of ruminal *Succiniclasticum* and *Ruminococcaceae UCG-014* in the GA, Gln, and AKG groups were all increased, with the GA group being significantly higher than the CON group (*p* < 0.05). Interestingly, the relative abundances of *Treponema 2* and *Prevotellaceae UCG-001* in the rumen of all three treatment groups were significantly lower than those in the CON group (*p* < 0.05) (Figure 2b).

**Figure 3:**
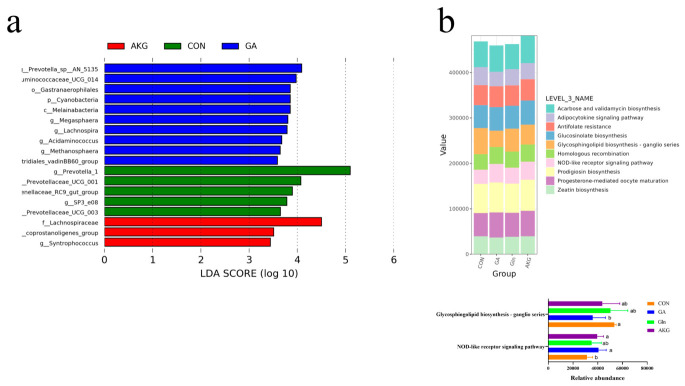
LEfSe analysis (**a**) and functional prediction (**b**) of ruminal bacteria in suckling lambs from the CON, GA, Gln, and AKG groups. CON: Each lamb was fed only milk replacer daily; GA: Based on the same milk replacer, each lamb received an additional 2 g/day of GA (Chengdu, China); AKG, Based on the same milk replacer, each lamb received an additional 2 g/day of AKG (Chengdu, China); Gln, Based on the same milk replacer, each lamb received an additional 2 g/day of Gln (Chengdu, China). In the bar charts, values with different letters differ significantly (*p* < 0.05).

LEfSe analysis revealed 18 taxa that differed significantly in rumen digesta. Among them, the CON group was significantly enriched with 5 bacterial species including *Prevotella 1* and *Prevotellaceae UCG-001*; the GA group was significantly enriched with 10 bacterial species including *Cyanobacteria* and *Lachnospira*; and the AKG group was significantly enriched with 3 bacterial species including *Lachnospiraceae* (Figure 3a). The metabolic pathways and functions of the rumen microbiota were predicted using PICRUSt2. At KEGG level 3, the metabolic pathways of the rumen microbiota in the four groups were predicted to be mainly enriched in pathways such as zeatin biosynthesis. Analysis of the metabolic pathways with relatively high abundance revealed that the NOD-like receptor signaling pathway in the CON group was predicted to be significantly lower than that in the GA and AKG groups, whereas the Glycosphingolipid biosynthesis—ganglio series pathway in the CON group was predicted to be significantly higher than that in the GA group (*p* < 0.05) (Figure 3b).

### 3.7. Effects of Glutamine and Its Precursors on the Composition and Diversity of Jejunal Microbiota in Suckling Lambs

**Figure 4:**
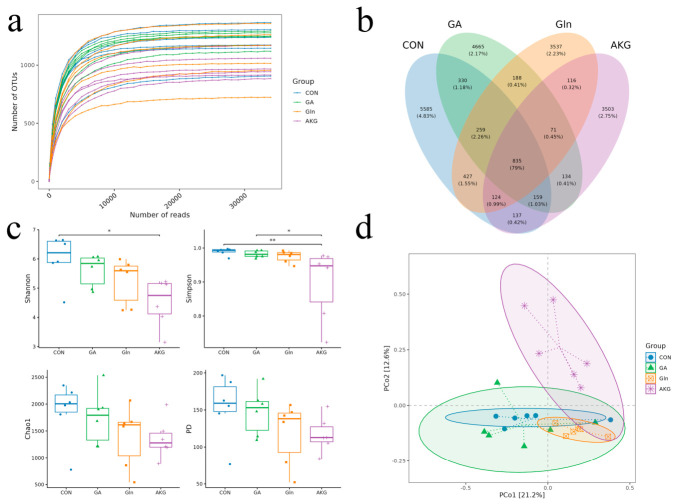
Rarefaction curves (**a**), OTUs (**b**), α-diversity analysis (**c**), and β-diversity analysis (**d**) of jejunal bacteria in suckling lambs from the CON, GA, Gln, and AKG groups. CON: Each lamb was fed only milk replacer daily; GA: Based on the same milk replacer, each lamb received an additional 2 g/day of GA (Chengdu, China); AKG, Based on the same milk replacer, each lamb received an additional 2 g/day of AKG (Chengdu, China); Gln, Based on the same milk replacer, each lamb received an additional 2 g/day of Gln (Chengdu, China). “*” indicates a significant difference between groups where *p* < 0.05. “**” indicates a significant difference between groups where *p* < 0.01.

As shown in Figure 4, the rarefaction curves for jejunal microbiota in all four groups approached asymptotes, indicating adequate sequencing depth that likely captured the vast majority of microbes present in the samples and may provide a reliable representation of the jejunal microbiota of the lambs (Figure 4a). Figure 4b shows that a total of 20,070 OTUs were identified across the four jejunal groups, of which 835 OTUs were shared, representing 4.16% of the total. The numbers of group-specific OTUs in the CON, GA, Gln, and AKG groups were 5585, 4665, 3537, and 3503, respectively. Alpha diversity analysis indicated that, compared with the CON group, the AKG group exhibited a significant reduction in both the Shannon and Simpson indices (*p* < 0.05) (Figure 4c). Principal coordinate analysis (PCoA) based on the Bray–Curtis dissimilarity matrix indicated a clear separation between the jejunal microbiota of the CON and AKG groups, reflecting substantial community-level differences (Figure 4d).

**Figure 5:**
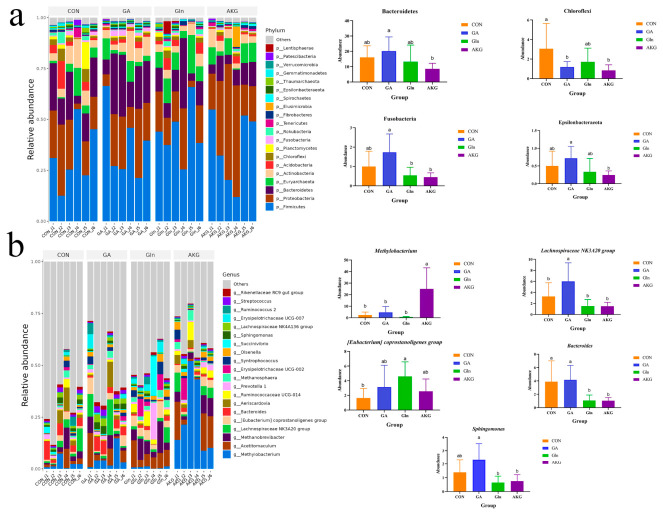
Relative abundance of jejunal bacteria at the phylum level (**a**) and genus level (**b**) in suckling lambs from the CON, GA, Gln, and AKG groups. CON: Each lamb was fed only milk replacer daily; GA: Based on the same milk replacer, each lamb received an additional 2 g/day of GA (Chengdu, China); AKG, Based on the same milk replacer, each lamb received an additional 2 g/day of AKG (Chengdu, China); Gln, Based on the same milk replacer, each lamb received an additional 2 g/day of Gln (Chengdu, China). In the bar charts, values with different letters differ significantly (*p* < 0.05).

At the phylum level, the dominant bacterial communities in the jejunum of all groups were Firmicutes, Proteobacteria, Bacteroidetes, and Euryarchaeota, with Firmicutes showing the highest relative abundance, followed by Proteobacteria. Among these, the relative abundance of Bacteroidetes in the GA group was significantly increased compared to the CON group (*p* < 0.05), but showed no significant difference from the Gln and AKG groups (*p* > 0.05). Compared with the CON group, the relative abundance of Chloroflexi in the AKG group was significantly reduced (*p* < 0.05). The relative abundances of Fusobacteria and Epsilonbacteraeota in the GA group were significantly higher than those in the AKG group (*p* < 0.05) (Figure 5a). At the genus level, the dominant bacterial communities in the jejunum of all groups were *Methylobacterium*, *Acetitomaculum*, *Methanobrevibacter*, and the *Lachnospiraceae NK3A20 group*. *Methylobacterium* had the highest relative abundance, followed by *Acetitomaculum*. Among these, the relative abundance of *Methylobacterium* in the AKG group was significantly higher than that in the CON, GA, and Gln groups (*p* < 0.05). The relative abundance of the *Lachnospiraceae NK3A20 group* in the GA group was significantly higher than that in the other three groups (*p* < 0.05). Compared with the CON group, the relative abundance of the *[Eubacterium] coprostanoligenes group* in the Gln group was significantly increased, while the relative abundances of *Bacteroides* and *Sphingomonas* in the Gln and AKG groups were significantly reduced (*p* < 0.05) (Figure 5b).

**Figure 6:**
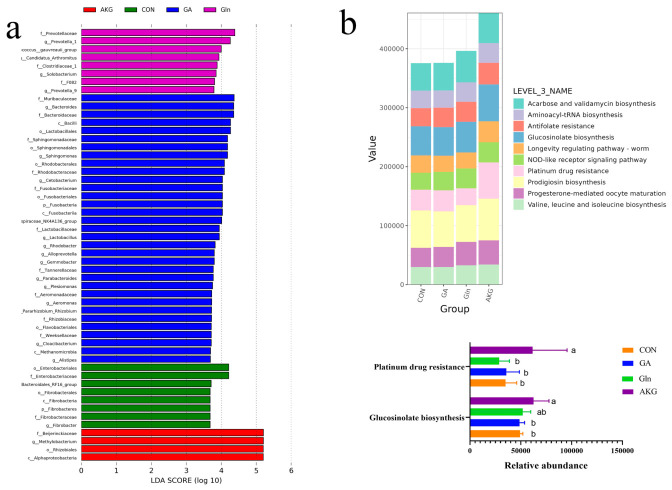
LEfSe analysis (**a**) and functional prediction (**b**) of jejunal bacteria in suckling lambs from the CON, GA, Gln, and AKG groups. CON: Each lamb was fed only milk replacer daily; GA: Based on the same milk replacer, each lamb received an additional 2 g/day of GA (Chengdu, China); AKG, Based on the same milk replacer, each lamb received an additional 2 g/day of AKG (Chengdu, China); Gln, Based on the same milk replacer, each lamb received an additional 2 g/day of Gln (Chengdu, China). In the bar charts, values with different letters differ significantly (*p* < 0.05).

LEfSe analysis identified 53 taxa that differed significantly in jejunal digesta. The CON, GA, Gln, and AKG groups were significantly enriched for 8, 33, 8, and 4 taxa, respectively (Figure 6a). PICRUSt2 was used to predict metabolic pathways and functions of the jejunal microbiota. At KEGG level 3, the metabolic pathways of the jejunal microbiota across the four groups were predicted to be primarily enriched in pathways such as valine, leucine and isoleucine biosynthesis. Analysis of pathways with relatively high relative abundance suggested that glucosinolate biosynthesis in the AKG group was predicted to be significantly higher than in the GA and CON groups, and platinum drug resistance in the AKG group was predicted to be significantly higher than in the other three groups (*p* < 0.05) (Figure 6b).

### 3.8. Effects of Glutamine and Its Precursors on Ileal Bacterial Composition and Diversity in Suckling Lambs

**Figure 7:**
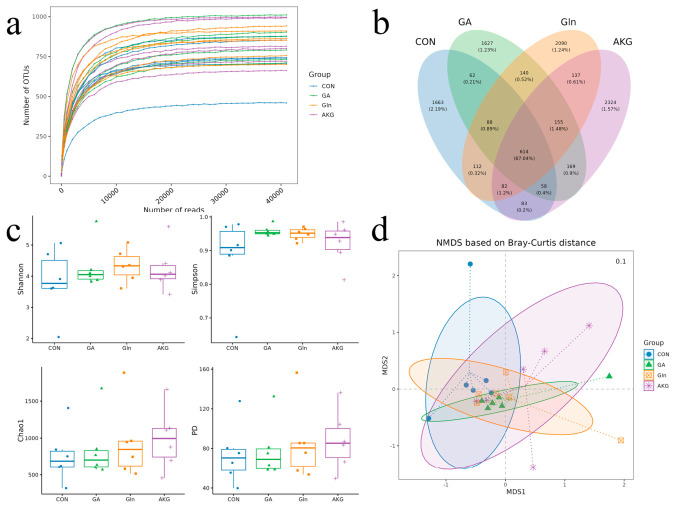
Rarefaction curves (**a**), OTUs (**b**), α-diversity analysis (**c**), and β-diversity analysis (**d**) of ileal bacteria in suckling lambs from the CON, GA, Gln, and AKG groups. CON: Each lamb was fed only milk replacer daily; GA: Based on the same milk replacer, each lamb received an additional 2 g/day of GA (Chengdu, China); AKG, Based on the same milk replacer, each lamb received an additional 2 g/day of AKG (Chengdu, China); Gln, Based on the same milk replacer, each lamb received an additional 2 g/day of Gln (Chengdu, China).

As shown in Figure 7, the rarefaction curves of ileal microbiota for all four groups tend toward asymptotes, indicating that the sequencing depth was adequate to capture the majority of microbial taxa present in the samples and may provide a reliable representation of the ileal microbiota of the lambs (Figure 7a). Figure 7b shows that a total of 9404 OTUs were identified across the four groups of suckling lambs, of which 614 OTUs were shared, accounting for 6.53% of the total. The numbers of group-specific OTUs in CON, GA, Gln, and AKG were 1663, 1627, 2090, and 2324, respectively. Alpha diversity analyses indicated no significant differences among the four groups in Shannon, Simpson, Chao1, and PD indices (*p* > 0.05) (Figure 7c). PERMANOVA (Bray–Curtis distance, 999 permutations) showed a significant difference in beta diversity of the ileal microbiota between the AKG group and the CON group (R^2^ = 0.08, *p* = 0.034) (Figure 7d).

**Figure 8:**
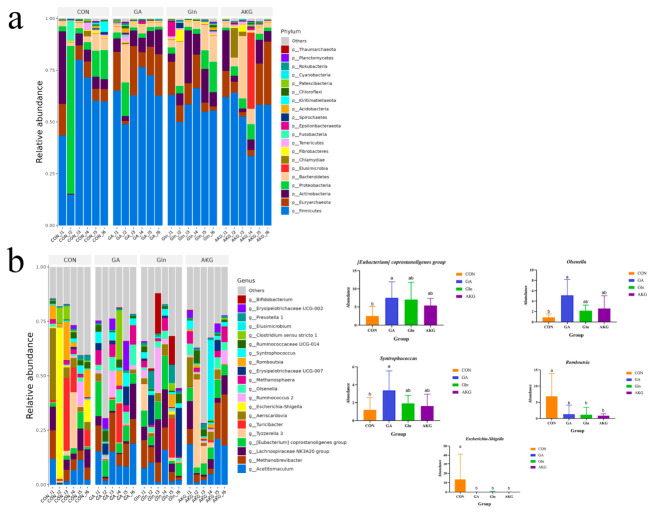
Relative abundance of ileal bacteria at the phylum level (**a**) and genus level (**b**) in suckling lambs from the CON, GA, Gln, and AKG groups. CON: Each lamb was fed only milk replacer daily; GA: Based on the same milk replacer, each lamb received an additional 2 g/day of GA (Chengdu, China); AKG, Based on the same milk replacer, each lamb received an additional 2 g/day of AKG (Chengdu, China); Gln, Based on the same milk replacer, each lamb received an additional 2 g/day of Gln (Chengdu, China). In the bar charts, values with different letters differ significantly (*p* < 0.05).

At the phylum level, the dominant bacterial communities in the ileum of all groups were Firmicutes, Euryarchaeota, Actinobacteria, and Proteobacteria, with Firmicutes showing the highest relative abundance, followed by Euryarchaeota. No significant differences in bacterial relative abundance were observed among the four groups (*p* > 0.05) (Figure 8a). At the genus level, the dominant bacterial communities in the ileum across all groups were *Acetitomaculum*, *Methanobrevibacter*, *Lachnospiraceae NK3A20 group*, and *[Eubacterium] coprostanoligenes group*, with *Acetitomaculum* having the highest relative abundance, followed by *Methanobrevibacter*. Among these, the GA group showed significant increases in the relative abundances of the *[Eubacterium] coprostanoligenes group*, *Olsenella*, and *Syntrophococcus* compared to the CON group (*p* < 0.05), but no significant differences were observed compared to the AKG and Gln groups (*p* > 0.05). The relative abundances of *Escherichia-Shigella* and *Romboutsia* in the CON group were significantly higher than those in the GA, Gln, and AKG groups (*p* < 0.05) (Figure 8b).

**Figure 9:**
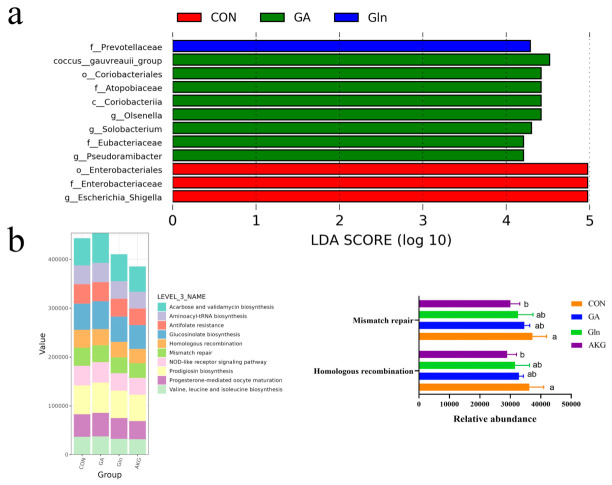
LEfSe analysis (**a**) and functional prediction (**b**) of ileal bacteria in suckling lambs from the CON, GA, Gln, and AKG groups. CON: Each lamb was fed only milk replacer daily; GA: Based on the same milk replacer, each lamb received an additional 2 g/day of GA (Chengdu, China); AKG, Based on the same milk replacer, each lamb received an additional 2 g/day of AKG (Chengdu, China); Gln, Based on the same milk replacer, each lamb received an additional 2 g/day of Gln (Chengdu, China). In the bar charts, values with different letters differ significantly (*p* < 0.05).

LEfSe analysis identified 12 taxa with significant differential abundance in ileal digesta. The CON, GA, and Gln groups were significantly enriched for 3, 8, and 1 bacterial taxa, respectively (Figure 9a). PICRUSt2 was used to predict metabolic pathways and functions of the ileal microbiota. At KEGG level 3, the metabolic pathways of the ileal microbiota across the four groups were predicted to be primarily enriched in pathways such as valine, leucine and isoleucine biosynthesis. Analysis of pathways with relatively high relative abundance revealed that homologous recombination and mismatch repair were predicted to be significantly higher in the CON group than in the AKG group (*p* < 0.05), whereas no significant differences were observed between the CON group and the GA or Gln groups (*p* > 0.05) (Figure 9b).

### 3.9. Correlation Analysis Between Differential Bacterial Genera in Rumen, Jejunal and Ileal Digesta and Growth Performance

**Figure 10:**
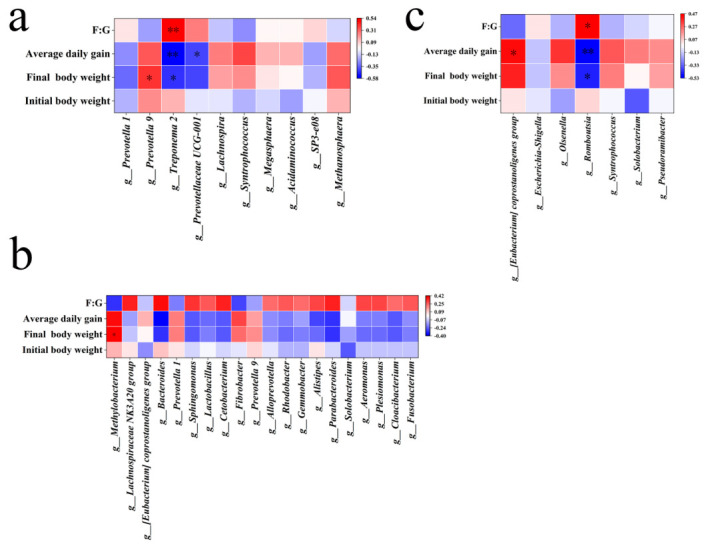
Correlation analysis between differential bacterial genera in rumen (**a**), jejunal (**b**) and ileal (**c**) digesta and growth performance. “*” indicates a significant difference between groups where *p* < 0.05. “**” indicates a significant difference between groups where *p* < 0.01. F:G, dry matter intake/average daily gain.

As shown in Figure 10, *Prevotella 9* in rumen digesta was significantly positively correlated with the final body weight of suckling lambs (*p* < 0.05), while *Treponema 2* was significantly negatively correlated with average daily gain and final body weight (*p* < 0.05) (Figure 10a). In jejunal digesta, *Methylobacterium* was significantly positively correlated with the final body weight of suckling lambs (*p* < 0.05) (Figure 10b). In ileal digesta, average daily gain was significantly positively correlated with the *[Eubacterium] coprostanoligenes group* (*p* < 0.05) and extremely significantly negatively correlated with *Romboutsia* (*p* < 0.01), while *Romboutsia* was significantly positively correlated with the F:G ratio (*p* < 0.05) (Figure 10c).

## 4. Discussion

Nutritional regulation during the suckling lamb stage has long been a central issue in ruminant production research. The lactation period represents one of the most rapid phases of lamb growth and development, with average daily gain and weaning weight directly affecting subsequent finishing efficiency and production returns. In the present study, supplementation with Gln, GA, and AKG each showed a tendency to increase final body weight and significantly increased average daily gain in suckling lambs, indicating that all three additives exert growth-promoting effects during the suckling stage. Nie et al. [29] reported that inclusion of rumen-protected glutamine in the diets of ewes during late gestation significantly increased lamb birth weight, 15-day weight, and average daily gain. Watford [30] noted that supplementation of sow and weanling pig diets with 0.5–1.0% Gln or GA during lactation and weaning improves intestinal and immune function and promotes superior growth performance. Moreover, Sun et al. [31] demonstrated that adding AKG at 500 and 1000 g/t to weanling pig diets significantly reduced diarrhoea incidence and improved nutrient digestibility and amino acid utilization, thereby enhancing overall piglet health. These studies are consistent with the present findings and collectively corroborate the growth-promoting effects of Gln, GA, and AKG in young animals. Glutamine serves as a primary energy substrate for enterocytes and lymphocytes, promoting villus development and crypt cell proliferation and thereby enhancing nutrient absorptive capacity [32]. Glutamic acid, as a precursor of glutathione and an oxidative substrate for the intestinal mucosa, indirectly supports intestinal barrier function [33]. AKG, as an intermediate of the tricarboxylic acid cycle, can stimulate protein synthesis and inhibit degradation via activation of the mTOR pathway, supplying metabolic fuel to the intestine [34,35]. Notably, in this study, the GA group exhibited a sustained increase in dry matter intake (DMI) throughout the experimental period, whereas increases in DMI in the AKG and Gln groups were observed only during days 21–42, suggesting that GA may promote feed intake through more direct effects on palatability or metabolic signalling. However, feed conversion ratio (F:G) did not differ significantly among treatments, indicating that the improvements in growth performance derive primarily from a combined effect of increased intake and nutrient utilization efficiency rather than from an isolated enhancement of feed conversion. Compared with monogastric species, suckling lambs are distinctive because the rumen is not yet fully developed; supplemented amino acids can bypass ruminal degradation and reach the small intestine directly, providing a unique early-life “window” for intervention with functional amino acids [33].

The antioxidant system of suckling lambs is not yet fully developed; their endogenous capacity to scavenge reactive oxygen species is limited, and their immune system remains immature, rendering them susceptible to the dual threats of oxidative stress and pathogenic infection. In animals, oxidative stress is a principal cause of cellular damage and disease development. The organism can mitigate hydroxyl radical formation and reduce oxidative injury by enhancing the activities of antioxidant enzymes such as CAT, SOD, and GSH-Px. GSH, as the central non-enzymatic antioxidant in animals, is synthesized from GA, cysteine, and glycine; Gln, GA, and AKG are all closely associated with the GSH biosynthetic pathway [36]. Moreover, Gln not only serves as a key energy substrate for rapidly proliferating cells such as lymphocytes and macrophages but also functions as an important regulator of the animal stress response, capable of enhancing antioxidant capacity and alleviating oxidative stress [37]. In the present study, supplementation with Gln, GA, and AKG each significantly increased serum CAT and GSH-Px activities in lambs, indicating that all three compounds can strengthen the antioxidant defenses of suckling lambs. Notably, the AKG group exhibited SOD activity significantly higher than that of the CON and Gln groups, while T-AOC was significantly increased in both the GA and AKG groups compared with the CON and Gln groups; concomitantly, MDA levels in the GA and AKG groups were significantly lower than in the CON group. These findings suggest that AKG is most effective at enhancing SOD activity, and that GA and AKG are comparable and superior to Gln in augmenting overall antioxidant capacity and reducing lipid peroxidation. GSH synthesis utilizes GA, cysteine, and glycine as direct precursors [36]. Exogenous GA can directly supply substrate to glutamate–cysteine ligase (GCL), promoting de novo GSH synthesis. Gln must first be hydrolyzed by glutaminase to GA before entering this pathway, and this conversion may be rate-limiting [30]; this limitation could partly explain why the improvements in T-AOC and MDA observed in the Gln group were inferior to those seen with GA and AKG in the present study. AKG exhibits a dual antioxidant mechanism: on one hand, AKG can be transaminated to GA and thereby indirectly contribute to GSH synthesis; on the other hand, AKG can directly react with H_2_O_2_ to produce succinate, water, and carbon dioxide while releasing ATP. This unique radical-scavenging pathway endows AKG with direct antioxidant capacity independent of the GSH pathway [38], which may underlie the significantly higher SOD activity observed in the AKG group in the present study. Wang et al. [39] reported that dietary supplementation with 1% Gln increased intestinal glutathione concentrations by 29% in weaned piglets, accompanied by improved growth performance. Gu et al. [40], using postpartum bovine fecal microbiota transplantation experiments, demonstrated that microbial metabolism of Gln, GA, glycine, and cysteine is closely related to GSH synthesis, suggesting that the antioxidant effects of exogenous Gln and GA may be partly mediated via modulation of gut microbial metabolism. Wang et al. [41] indicated that periparturient dairy cows supplemented with AKG (5 g and 10 g/day) exhibited significantly elevated serum GSH-Px levels. These previous findings are consistent with the results of the present study. Antioxidant function is closely linked to immune competence. GSH not only directly neutralizes free radicals but also influences lymphocyte proliferation and differentiation [42]. In this study, serum IgA and IgG concentrations in the GA, AKG, and Gln groups were all significantly higher than those in the CON group, indicating that all three additives effectively enhanced humoral immunity in suckling lambs. Additionally, only the GA group showed a significant increase in IgM compared with the CON group. At the cytokine level, compared with the CON group, IL-1β concentrations were significantly reduced in the GA and AKG groups, whereas the Gln group did not differ significantly from CON; IL-6 concentrations were significantly lower in the GA, AKG, and Gln groups compared with the CON group. Notably, IL-10 concentrations were significantly higher in the CON group than in the AKG group, whereas GA and Gln groups did not differ significantly from CON. These results indicate that GA and AKG appear to be more effective than Gln at suppressing pro-inflammatory cytokines IL-1β and IL-6, while AKG displayed a tendency to reduce the anti-inflammatory cytokine IL-10. Ruth and Field [43] systematically delineated the immunoregulatory effects of amino acids on gut-associated lymphoid tissue (GALT), indicating that specific amino acids such as Gln, GA, and arginine are critical for maintaining intestinal integrity, promoting intestinal growth and function, and optimizing GALT immune responses, including modulation of proinflammatory cytokine secretion and promotion of plasma cell IgA secretion. Cruzat et al. [11] noted that Gln is an important metabolic substrate for rapidly proliferating cells such as lymphocytes and macrophages; under immunological stress, systemic demand for Gln increases markedly, and supplementation with exogenous Gln can support immune cell proliferation and function. In the present study, the Gln group exhibited significant increases in IgA and IgG and a significant decrease in IL-6, consistent with the expected effects of Gln in supporting humoral immunity and attenuating certain inflammatory responses. GA in this study demonstrated a more comprehensive immunomodulatory profile, significantly increasing IgA, IgG, and IgM while markedly reducing IL-1β and IL-6. This outcome may relate to GA’s central role in GSH synthesis and antioxidant defense. As a precursor for GSH synthesis, GA can indirectly mitigate inflammatory responses by enhancing the organism’s antioxidant capacity [44]. The immunomodulatory effects of AKG observed here were more complex. On one hand, AKG significantly increased IgA and IgG and reduced IL-1β and IL-6, showing anti-inflammatory and humoral immune-promoting effects similar to GA; on the other hand, IL-10 levels in the AKG group were significantly lower than those in the control group, a result inconsistent with Wang et al. [41], who reported elevated blood IL-10 following AKG supplementation in periparturient dairy cows. Potential reasons for this discrepancy include interspecies differences, differences in physiological stage, dosage variations, and modes of supplementation. In summary, Gln primarily appears to enhance humoral immunity (IgA, IgG) and suppresses IL-6, with relatively modest antioxidant effects; GA appears to enhance all three classes of immunoglobulins while significantly suppressing IL-1β and IL-6 and effectively improving T-AOC and MDA, suggesting a relatively comprehensive-antioxidant synergy; AKG is comparable to GA in enhancing IgA and IgG and suppressing IL-1β and IL-6, and it is particularly notable for improving SOD and T-AOC, though its suppressive effect on IL-10 warrants further investigation.

The lactation period represents a critical window for the establishment of gastrointestinal microbiota in lambs. The intestinal microbial community structure of neonatal lambs undergoes dramatic changes after birth; with the rumen not yet fully developed, the composition and metabolic functions of the gut microbiota directly influence the host’s subsequent growth performance and health status [45]. Gln, GA, and AKG are not only important energy substrates for intestinal epithelial cells but can also be directly utilized by gut microbes or metabolically converted into active compounds such as short-chain fatty acids, thereby modulating microbial composition and host health [46,47]. In this study, the three additives produced pronounced differences in microbial diversity across intestinal segments of suckling lambs. In the rumen, the GA group exhibited significantly lower Shannon and Simpson indices compared with the CON group; in the jejunum, the AKG group showed significantly reduced Shannon and Simpson indices relative to the CON group; in the ileum, none of the α-diversity indices differed significantly among the four groups. Beta diversity analysis suggested that the ruminal microbiota of the GA, Gln, and AKG groups did not completely overlap with that of the CON group. In the jejunum, the microbial communities of the CON and AKG groups formed separate clusters, and in the ileum, only the AKG group exhibited a discernible difference from the CON group. These findings indicate site-specific responses of gastrointestinal segments to amino acid supplementation, with the rumen and jejunum microbiota being more responsive to the additive interventions, which may be attributable to the higher microbial abundance and greater metabolic activity in these two sites [48,49]. The results of the present study are highly consistent with those of previous investigations into microbial community composition. Liao et al. [50] systematically reviewed the regulatory effects of GA and Gln on the gut microbiota, noting that GA and Gln supplementation can elevate levels of beneficial bacteria and suppress pathogenic bacteria; thus, modulating the gut microbial ecosystem through nutritional strategies is an effective approach to optimize intestinal health and function. At the phylum level, the relative abundance of Bacteroidetes in the rumen was significantly reduced in the GA group; the relative abundance of Spirochaetes in the rumen was significantly reduced in the GA, Gln, and AKG groups, and because Spirochaetes include some potential pathogens (e.g., *Treponema*), their reduction is favorable for host health. At the genus level, the relative abundances of *Succiniclasticum* and *Ruminococcaceae UCG-014* were significantly higher in the GA group than in the CON group. Ruminococcaceae is a well-known butyrate-producing family, and the increase in its abundance is highly consistent with Chen et al. [51], who reported that Gln and GA can increase Ruminococcaceae abundance and stimulate butyrate production. The potential pathogens *Treponema 2* and *Prevotellaceae UCG-001* were significantly reduced in all three treatment groups, and LEfSe analysis also revealed taxa significantly enriched in each of the four groups, further confirming the overall reshaping effect of amino acid supplementation on the microbial ecosystem. In the jejunum, the relative abundance of Bacteroidetes contrasted with the trend observed in the rumen, reflecting segment-specific microbial responses to the same additive. Notably, in the ileum, the relative abundance of the pathogenic genus *Escherichia-Shigella* in the CON group was significantly higher than in the GA, Gln, and AKG groups. *Escherichia-Shigella* comprises common enteric pathogens such as *Escherichia coli* and *Shigella* spp., which are important etiological agents of diarrhea in young animals. This finding is consistent with several previous studies [51,52,53,54]. All three additives significantly reduced the abundance of this genus, suggesting a potential role in limiting the colonization of potentially pathogenic bacteria. However, it should be noted that 16S rRNA gene sequencing alone cannot confirm pathogenicity in this study, and further functional studies (e.g., metagenomics or culturing) are needed to validate our findings. The regulatory effect of AKG on gut bacteria was also evident in this study. The relative abundance of *Methylobacterium* in the jejunum of the AKG group was significantly higher than in the other three groups. The correlation analysis in this study also confirmed that an increased relative abundance of *Methylobacterium* is associated with gains in lamb body weight. In the jejunum of the GA group, the relative abundance of *Lachnospiraceae NK3A20 group* was significantly higher than in the other three groups, and in the ileum of the GA group, the relative abundances of *[Eubacterium] coprostanoligenes group*, Olsenella, and Syntrophococcus were significantly increased. The studies by Chen et al. [55] and Jiang et al. [56] provide important support for AKG-mediated modulation of the microbiota. PICRUSt2 functional prediction analysis further elucidated alterations in microbial metabolic functions. In the rumen, the abundance of the NOD-like receptor signaling pathway in the CON group was significantly lower than in the GA and AKG groups; this pathway is critical for host recognition of intracellular pathogenic microbes and initiation of immune responses, and its predicted increase in abundance may be associated with immune modulation induced by shifts in microbial composition [47,57]. In the jejunum, the AKG group exhibited significantly higher abundances of the glucosinolate biosynthesis and platinum drug resistance pathways compared with the other groups. In the ileum, DNA repair pathways (homologous recombination and mismatch repair) were significantly more abundant in the CON group than in the AKG group, which may reflect differences in levels of oxidative stress faced by the microbiota under the different treatments. Overall, in this study, the effects of GA and Gln on microbial composition were generally stronger than those of AKG, consistent with Vanden Abbeele et al. [47], who reported that GA and Gln selectively promote the enrichment of butyrate-producing bacteria, whereas the effects of AKG are manifested more in the modulation of broader microbial taxa [51]. Two explanations may account for this difference. First, from the perspective of microbial utilization, GA and Gln can be directly metabolized by gut microbes as nitrogen and carbon sources, whereas AKG must first be converted by microbial enzymatic systems (e.g., aminotransferases) into Gln or GA before entering similar metabolic pathways; this conversion may be rate-limiting or taxon-specific. Second, from a functional targeting standpoint, microbial metabolites of GA and Gln directly supply energy to intestinal epithelial cells and modulate immune functions, whereas AKG-derived metabolites more readily enter the host tricarboxylic acid cycle, so their effects on the microbiome may depend more on indirect mechanisms. Last but not least, the significant increases in ALB induced by Gln, GA, and AKG in this study align with previous findings for other functional amino acids [34,58,59,60], which may reflect their direct promotion of hepatic protein synthesis. Gln serves as an important substrate for GSH and the synthesis of multiple functional amino acids; GA is a direct precursor for GSH synthesis; and AKG can promote protein synthesis by modulating the tricarboxylic acid cycle and mTOR signaling. However, none of the three compounds produced significant effects on other biochemical indices, including TP, GLB, TC, and TG, suggesting that under the conditions of this study, supplementation with Gln and its precursors did not induce aberrant fluctuations in lipid metabolism, hepatic or renal function, or glucose metabolism, which reflects favorable metabolic compatibility and safety. One limitation of this study is the moderate sample size for microbiota analysis (*n* = 6 per group), which may reduce the power to detect small effect sizes. Future studies with larger cohorts are needed to confirm our findings.

## 5. Conclusions

Results obtained from the current study demonstrated that daily 2 g supplementation of Gln, GA, or AKG in suckling lambs significantly improved growth performance, serum antioxidant and immune functions, and differentially modulated gut microbiota. GA increased dry matter intake throughout the trial, while AKG and Gln acted only in the later phase during the experimental period. AKG showed the strongest antioxidant effect, and GA was superior in immune modulation. All three additives reduced potential pathogens, with GA enriching ruminal butyrate-producing taxa. These functional amino acids are safe and effective nutritional strategies for the health of lambs during the suckling period, providing a theoretical basis for their application in young ruminants. Future studies with larger sample sizes and dose–response designs are needed to further validate these findings.

## Figures and Tables

**Table 1:** Composition and nutrient levels of the starter (DM basis).

Ingredients, %	
Corn	55.80
Soybean meal	20.20
Cottonseed mean	18.70
NaCl	0.30
NaHCO_3_	0.60
CaHPO_4_	0.40
Premix ^1^	1.00
Total	100.00
Nutrient levels	
Dry matter	97.12
Crude protein	18.72
Ether extract	5.40
Neutral detergent fiber	21.67
Acid detergent fiber.	8.11
Calcium	0.78
Phosphorus	0.41
Metabolic energy ^2^, MJ/kg	17.62

^1^ The premix provided following per kilogram of diet: Fe 280 mg, Zn 50 mg, Mn 40 mg, Cu 10 mg. I 0.60 mg, Se 0.40 mg, Co 0.20 mg, VA 8000 IU, VD 900 IU, VE 30 IU. ^2^ ME was a calculated value; the other nutritional levels were measured values.

**Table 2:** Effects of glutamine and its precursors on the growth performance of suckling lambs (*n* = 10/group).

Items	Groups				*p*-Value
	CON	GA	AKG	Gln	
Body weight (kg)					
Initial BW	7.22 ± 1.31	7.47 ± 1.24	7.44 ± 1.35	7.34 ± 1.12	0.978
Final BW	14.77 ± 1.83 ^b^	17.32 ± 2.12 ^a^	17.69 ± 1.84 ^a^	17.73 ± 3.38 ^a^	0.056
ADG (g/d)	179.76 ± 43.05 ^b^	234.52 ± 29.17 ^a^	244.20 ± 27.22 ^a^	247.47 ± 68.64 ^a^	0.019
DMI (g/head/d)					
T1 (Day 1 to 10)	217.77 ± 27.05 ^bc^	251.64 ± 38.11 ^a^	192.92 ± 25.60 ^c^	226.87 ± 27.26 ^ab^	0.001
T2 (Day 11 to 20)	343.3 ± 32.26 ^b^	417.56 ± 65.34 ^a^	354.41 ± 72.04 ^b^	367.45 ± 64.50 ^ab^	0.047
T3 (Day 21 to 30)	529.83 ± 51.44 ^b^	683.95 ± 78.54 ^a^	626.55 ± 44.63 ^a^	640.75 ± 89.83 ^a^	<0.001
T4 (Day 31 to 42)	719.81 ± 51.18 ^b^	876.52 ± 53.99 ^a^	853.37 ± 99.15 ^a^	852.35 ± 62.12 ^a^	<0.001
F:G	2.64 ± 0.78	2.38 ± 0.30	2.09 ± 0.25	2.35 ± 1.13	0.498

DMI, dry matter intake; ADG, average daily gain; CON: Each lamb was fed only milk replacer daily; GA: Based on the same milk replacer, each lamb received an additional 2 g/day of GA (Chengdu, China); AKG, Based on the same milk replacer, each lamb received an additional 2 g/day of AKG (Chengdu, China); Gln, Based on the same milk replacer, each lamb received an additional 2 g/day of Gln (Chengdu, China). In the same row, values with different superscripts differ significantly (*p* < 0.05). F:G, DMI/ADG.

**Table 3:** Effects of glutamine and its precursors on serum immunoglobulin levels in suckling lambs (*n* = 8/group).

Items	Groups				*p*-Value
	CON	GA	AKG	Gln	
IgA (g/L)	0.67 ± 0.06 ^b^	0.74 ± 0.05 ^a^	0.73 ± 0.06 ^a^	0.73 ± 0.06 ^a^	0.024
IgG (g/L)	15.05 ± 0.93 ^b^	17.51 ± 2.38 ^a^	18.09 ± 1.57 ^a^	18.44 ± 1.55 ^a^	0.002
IgM (g/L)	1.06 ± 0.19 ^b^	1.36 ± 0.35 ^a^	1.26 ± 0.27 ^ab^	1.15 ± 0.25 ^ab^	0.043

IgA: immunoglobulin A; IgG: immunoglobulin G; IgM: immunoglobulin M; CON: Each lamb was fed only milk replacer daily; GA: Based on the same milk replacer, each lamb received an additional 2 g/day of GA (Chengdu, China); AKG, Based on the same milk replacer, each lamb received an additional 2 g/day of AKG (Chengdu, China); Gln, Based on the same milk replacer, each lamb received an additional 2 g/day of Gln (Chengdu, China). In the same row, values with different superscripts differ significantly (*p* < 0.05).

**Table 4:** Effects of glutamine and its precursors on blood inflammatory cytokines in suckling lambs (*n* = 8/group).

Items	Groups				*p*-Value
	CON	GA	AKG	Gln	
TNF-α (pg/mL)	323.88 ± 124.65	236.38 ± 62.76	242.94 ± 57.69	277.31 ± 96.19	0.211
IL-1β (pg/mL)	81.25 ± 23.47 ^a^	52.00 ± 31.98 ^b^	49.75 ± 24.72 ^b^	61.88 ± 27.74 ^ab^	0.046
IL-6 (pg/mL)	176.13 ± 52.20 ^a^	119.25 ± 34.90 ^b^	111.44 ± 34.91 ^b^	116.13 ± 43.46 ^b^	0.014
IL-10 (pg/mL)	103.27 ± 12.69 ^a^	98.05 ± 9.67 ^a^	85.55 ± 11.50 ^b^	92.25 ± 11.50 ^ab^	0.026

TNF-α: tumor necrosis factor-α; IL-1β: interleukin-1β; IL-6: interleukin-6; IL-10: interleukin-10; CON: Each lamb was fed only milk replacer daily; GA: Based on the same milk replacer, each lamb received an additional 2 g/day of GA (Chengdu, China); AKG, Based on the same milk replacer, each lamb received an additional 2 g/day of AKG (Chengdu, China); Gln, Based on the same milk replacer, each lamb received an additional 2 g/day of Gln (Chengdu, China). In the same row, values with different superscripts differ significantly (*p* < 0.05).

**Table 5:** Effects of glutamine and its precursors on the antioxidant capacity of blood in suckling lambs (*n* = 8/group).

Items	Groups				*p*-Value
	CON	GA	AKG	Gln	
SOD (U/mL)	80.72 ± 4.48 ^c^	87.50 ± 3.47 ^ab^	90.63 ± 4.51 ^a^	85.52 ± 2.36 ^b^	0.001
CAT (U/mL)	1.77 ± 0.77 ^b^	2.95 ± 1.14 ^a^	2.89 ± 0.95 ^a^	3.02 ± 1.01 ^a^	0.029
GSH-Px (U/mL)	162.11 ± 10.80 ^c^	180.77 ± 5.49 ^a^	172.80 ± 3.80 ^ab^	169.64 ± 13.88 ^b^	0.005
T-AOC (mmol/L)	0.48 ± 0.07 ^b^	0.60 ± 0.04 ^a^	0.64 ± 0.08 ^a^	0.52 ± 0.03 ^b^	0.001
MDA (μmol/L)	4.82 ± 0.39 ^a^	4.08 ± 0.51 ^b^	4.05 ± 0.57 ^b^	4.50 ± 0.25 ^ab^	0.005

SOD: superoxide dismutase; CAT: catalase; GSH-Px: glutathione peroxidase; T-AOC: total antioxidant capacity; MDA: malondialdehyde; CON: Each lamb was fed only milk replacer daily; GA: Based on the same milk replacer, each lamb received an additional 2 g/day of GA (Chengdu, China); AKG, Based on the same milk replacer, each lamb received an additional 2 g/day of AKG (Chengdu, China); Gln, Based on the same milk replacer, each lamb received an additional 2 g/day of Gln (Chengdu, China). In the same row, values with different superscripts differ significantly (*p* < 0.05).

**Table 6:** Effects of glutamine and its precursors on blood biochemical parameters in suckling lambs (*n* = 8/group).

Items	Groups				*p*-Value
	CON	GA	AKG	Gln	
TP (g/L)	56.16 ± 5.74	56.51 ± 4.78	55.03 ± 4.29	52.69 ± 5.30	0.44
ALB (g/L)	24.58 ± 2.29 ^b^	28.00 ± 2.01 ^a^	27.27 ± 2.51 ^a^	27.06 ± 2.74 ^a^	0.044
GLB (g/L)	31.58 ± 6.45	28.51 ± 4.68	27.75 ± 4.76	25.64 ± 3.54	0.142
TC (mmol/L)	0.77 ± 0.30	0.64 ± 0.18	0.89 ± 0.27	0.77 ± 0.27	0.333
TG (mmol/L)	0.38 ± 0.19	0.29 ± 0.15	0.37 ± 0.17	0.30 ± 0.16	0.631
BUN (mg/dL)	16.70 ± 5.98	22.40 ± 7.59	19.66 ± 3.21	19.50 ± 5.22	0.287
ALP (U/L)	596.74 ± 400.51	927.45 ± 465.41	728.17 ± 347.34	842.14 ± 462.33	0.421
AST (U/L)	148.62 ± 96.27	183.77 ± 126.65	152.79 ± 102.44	201.40 ± 172.43	0.816
ALT (U/L)	20.84 ± 22.05	17.22 ± 2.92	23.07 ± 17.54	15.27 ± 3.00	0.694
GLU (mmol/L)	4.84 ± 0.46	5.20 ± 0.41	5.27 ± 0.69	5.21 ± 0.45	0.338

TP, total protein; ALB, albumin; GLB, globulin; TC, total cholesterol; TG, triglyceride; BUN, blood urea nitrogen; ALP, alkaline phosphatase; AST, aspartate transaminase; ALT, alanine transaminase; GLU, glucose; CON: Each lamb was fed only milk replacer daily; GA: Based on the same milk replacer, each lamb received an additional 2 g/day of GA (Chengdu, China); AKG, Based on the same milk replacer, each lamb received an additional 2 g/day of AKG (Chengdu, China); Gln, Based on the same milk replacer, each lamb received an additional 2 g/day of Gln (Chengdu, China). In the same row, values with different superscripts differ significantly (*p* < 0.05).

## Data Availability

The sequences from the current study have been deposited in the NCBI Sequence Read Archive database with the accession number PRJNA1467527; other data are available from the corresponding author.
